# Deep Subwavelength-Scale Light Focusing and Confinement in Nanohole-Structured Mesoscale Dielectric Spheres

**DOI:** 10.3390/nano9020186

**Published:** 2019-02-01

**Authors:** Yinghui Cao, Zhenyu Liu, Oleg V. Minin, Igor V. Minin

**Affiliations:** 1 College of Computer Science and Technology, Jilin University, 2699 Qianjin Street, Changchun 130012, China; caoyh@jlu.edu.cn; 2 Changchun Institute of Optics, Fine Mechanics and Physics, 3888 East Nanhu Road, Changchun 130033, China; liuzy@ciomp.ac.cn; 3 Radiohpysical Department, Tomsk State University, 30 Lenin Avenue, Tomsk 634050, Russia; oleg.minin@ngs.ru; 4 Nondestructive Testing School, Tomsk Polytechnic University, 36 Lenin Avenue, Tomsk 634050, Russia

**Keywords:** nanohole, microsphere, subwavelength-scale light focusing

## Abstract

One of the most captivating properties of dielectric mesoscale particles is their ability to form a sub-diffraction limited-field localization region, near their shadow surfaces. However, the transverse size of the field localization region of a dielectric mesoscale particle is usually larger than λ/3. In this present paper, for the first time, we present numerical simulations to demonstrate that the size of the electromagnetic field that forms in the localized region of the dielectric mesoscale sphere can be significantly reduced by introducing a nanohole structure at its shadow surface, which improves the spatial resolution up to λ/40 and beyond the solid immersion diffraction limit of λ/2*n*. The proposed nanohole-structured microparticles can be made from common natural optical materials, such as glass, and are important for advancing the particle-lens-based super-resolution technologies, including sub-diffraction imaging, interferometry, surface fabrication, enhanced Raman scattering, nanoparticles synthesis, optical tweezer, etc.

## 1. Introduction

Photonic Nanojet (PNJ) is the phenomenon of subwavelength-scale light focusing that is generated by dielectric microparticles. It has been widely applied in different areas, including laser cleaning, nanolithography, super-resolution imaging, enhanced Raman scattering, non-linear fluorescence enhancement, etc. [1,2,3,4]. However, one limitation for PNJ is its minimum beam width of λ/3 [1] (where λ is the wavelength of the incident light) and, thus, more efforts are needed to further reduce the focal spot sizes of the PNJ.

Over the past decade, many studies have been focused on decreasing the width of the PNJ to be as small as possible. If this could be achieved, PNJs would be able to provide new pathways for the trapping of nanoparticles and cells [5,6], subwavelength optical imaging [7], ultrafast all-optical switching [8], etc. Multilayer [9] and a two-layer [10] graded refractive index dielectric particles have also been considered. By using the anomalously intensity-enhanced apodization effect, PNJ with a Full Width at Half Maximum (FWHM) focal spot size that is less than 0.3λ have been attained in previous studies [11,12]. PNJ with a FWHM focal spot size of 0.29λ, which was generated by overstepping the upper refractive index limit has also been reported in the literature [13].

Nanohole-structured dielectric objects have also been considered in the literature. For example, as shown in Reference [14], the effects of the total light far-field scattering of deep holes in the spherical particles with a refractive index near 1.05, were investigated, which showed that these structural details had a negligible influence. In Reference [15], it was shown that the PNJ beam sizes shrank by nearly 28%, due to the introducing of concentric rings on the illumination side of microspheres. To control the unidirectional scattering by the spectral overlapping of the Mie-type resonant modes, a dielectric high-index nanocylinder (n ~ 3.4), with an axial nanohole, has been considered in some studies [16]. Nevertheless, although several methods of light localization have been investigated, deep subwavelength localization, based on a single dielectric mesoscale particle, has not yet been realized.

In this work, a nanohole-structured dielectric microsphere is proposed for deep subwavelength-scale light focusing and strong light confinement, well below the diffraction limit. The field enhancement from the nanostructured mesoscale dielectric microsphere is due to the permittivity contrast between the material of the microsphere and the nanohole. The proposed nanohole-structured microparticles can be made from common dielectric materials, such as glass or latex, and has the advantage of the ability to tailor the spatial region of light confinement and enhancement by choosing the proper geometry, shape, and size of the nanohole. The approach provides a wide platform for deep subwavelength focusing and imaging, which offers the capability of sub-diffraction techniques for microscopy systems, light–matter interactions, interferometry, surface fabrication, enhanced Raman scattering, optical tweezer, and information processing.

This paper has been organized as follows. In Section 2, microspheres without a nanohole have been numerically modeled and analyzed. In Section 3, nanohole-structured microspheres have been simulated and analyzed. Finally, in Section 4, conclusions have been drawn.

## 2. Dielectric Microspheres without Hole

**Figure 1:**
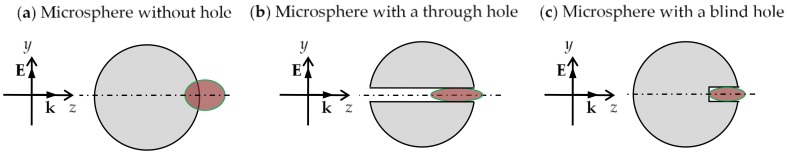
Schematic diagrams for the simulated dielectric sphere (**a**) without a nanohole, (**b**) with a through hole, and (**c**) with a blind hole. The incident light is a plan wave that propagates along the z-axis, **k** is the wave vector. The incident field **E** is polarized along the y-axis. The oval-shaped zones in dark red color indicate the focal spots, and dash-dotted lines indicate the symmetrical axes for the spheres.

This section describes the dielectric microspheres with a fixed refractive index of *n* = 1.5 and different sphere diameters of *D_s_* = 1.5λ, 2.5λ, 3.5λ and 4.5λ, which were numerically modeled and simulated. For λ = 600 nm, the simulated dielectric spheres had a diameter of *D_s_* = 0.9, 1.5, 2.1, and 2.7 µm, respectively. We selected a refractive index of *n* =1.5, because in an optical band, many commonly used dielectric materials have a refractive index nearly equal to 1.5, such as glass, PMMA, fused silica, etc. [17]. We chose the popular spherical shape of a microparticle for the photonic nanojet formation [4]. The dielectric microspheres were modeled and simulated by using the commercial software COMSOL Multiphysics, which is based on the finite elements method (FEM). In this simulation, a non-uniform mesh was employed to reduce the computational cost and the Perfect Matched Layer (PML) was applied as the boundary condition. The incident light was assumed to be a plane wave that propagates along the z-axis, with a linear polarization along the y-axis. A schematic diagram for the simulated dielectric microsphere is given in Figure 1a.

**Figure 2:**
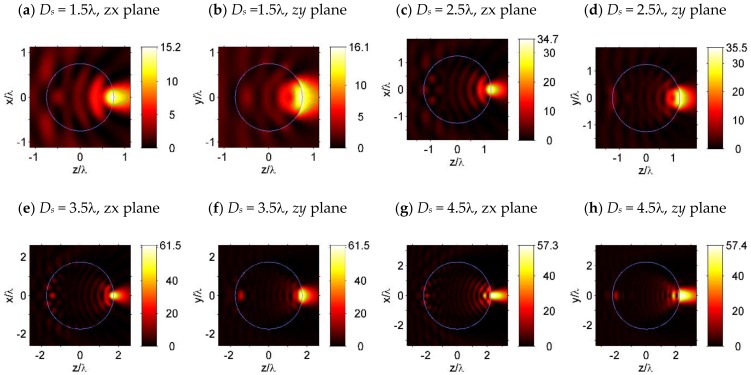
Light intensity distribution of a simulated microsphere with a sphere diameter of (**a**,**b**) *D_s_* = 1.5λ, (**c**,**d**) *D_s_* = 2.5λ, (**e**,**f**) *D_s_* = 3.5λ, and (**g**,**h**) *D_s_* = 4.5λ. Subfigures (**a**,**c**,**e**,**g**) are plotted in the zx-plane, which is perpendicular to the plane of polarization; subfigures (**b**,**d**,**g**), and (**h**) are plotted in the zx-plane, which is the plane of polarization. The Full Width at Half Maximum (FWHM) focal spot of the simulated microspheres are indicated by the contour lines at the value of half maximum light intensity 0.5*I_max_*, which are plotted by the solid green lines.

Figure 2 shows the light intensity distribution around the simulated dielectric spheres. The light focusing properties of the simulated microspheres are shown in Table 1, including the FWHM focal spot sizes along the *x*, *y*, and *z*-axis (*S_x_*, *S_y_*, *S_z_*), the focal volumes *V* (which were obtained by volume integration inside the FWHM focal spot) and the maximum light intensity *I_max_*, in the focal spot. After combining Figure 2 and Table 1, we found that, with an increase in the sphere diameter from *D_s_* = 1.5λ to 3.5λ, the focal volume *V* decreased from 0.057λ^3^ to 0.051λ^3^, while the maximum light intensity *I_max_* increased from 16.1*I*_0_ to 61.1*I*_0_ (*I*_0_ is the light intensity of the incident light). Furthermore, when the sphere diameter was increased to *D_s_* = 4.5λ, the focal spot split into two parts, with the major part of the focal spot expanding into the surrounding medium, and a smaller part staying inside the microsphere [2,4]. Thus, for the microspheres with a refractive index of *n* = 1.5, when the sphere had a smaller diameter (*D_s_* < 4.5λ), the light incident on the sphere was focused on the shadow surface (the surface of the particle that is opposite to the irradiated side [4]) of the sphere. When the sphere was large enough (*D_s_* ≥ 4.5λ), PNJ formed beyond the shadow surface of the sphere [2,4], and the second focal spot formed inside the sphere and close to its shadow surface, due to light reflection at the inner side of the shadow surface [4]. It could be noted that, in this case, the maximal field intensity was slightly reduced (Table 1).

## 3. Nanohole-Structured Dielectric Microspheres

This section describes the nanohole-structured microspheres with a sphere diameter of *D_s_* = 3.5λ and a refractive index of *n* = 1.5, which were simulated and analyzed. To make the structure clear, schematic diagrams for the simulated microspheres with a through hole and a blind hole are depicted in Figure 1b,c, respectively.

### 3.1. Microspheres with a through Hole

**Figure 3:**
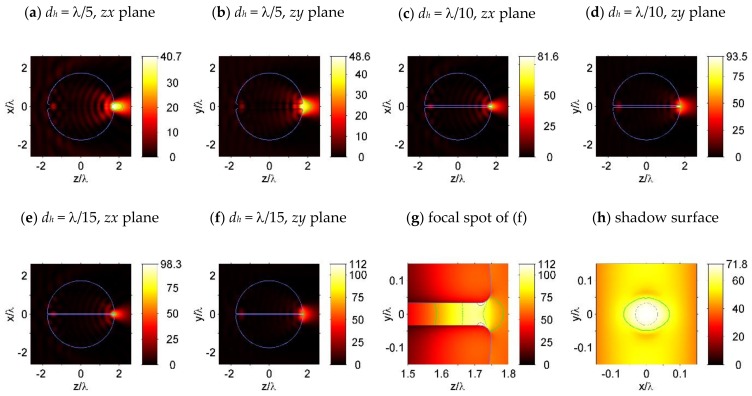
Light intensity (|E|^2^) of the simulated dielectric microspheres with a nanohole of size (**a**,**b**) *d_h_* = λ/5, (**c**,**d**) *d_h_* = λ/10, and (**e**–**h**) *d_h_* = λ/15. The sphere diameter and refractive index are set as *D_s_* = 3.5λ and *n* = 1.5. Features of the focal spot in (**f**) are plotted in (**g**), where the green solid lines, the gray dashed lines, and the gray solid lines indicate the contour lines at values 0.5*I_max_*, 0.8*I_max_*, and 0.9*I_max_,* respectively. For a sphere with a hole size of *d_h_* = λ/15, light intensity along a plane that is 0.001λ from the shadow surface is plotted in (**h**), with a circle plotted in gray dashed lines, indicating the projection of the hole interface.

First, the nanohole-structured microsphere with a through hole of diameter *d_h_* = λ/5, λ/10, and λ/15 were simulated; light intensity around the simulated microspheres are shown in Figure 3. The corresponding focal spot properties, including the FWHM focal spot sizes (*S_x_*, *S_y_*, *S_z_*), the focal volumes *V*, and the maximum light intensity *I_max_*, in the focal spot, are shown in Table 2. After comparing Figure 3a,b with Figure 2e,f, we found that the focal spot sizes (*S_x,y,z_*) and focal volume *V* of the λ/5-nanohole-structured microsphere were even larger than that of the microspheres without a nanohole, which could be explained as the weakening of the light focusing capability of the dielectric microsphere, due to the comparatively large λ/5-sized hole. When the hole size was reduced to be smaller than λ/10, the focal spot sizes and focal volume were reduced considerably, as shown in Figure 3c–h and Table 2. Finally, the features of the FWHM focal spot of the microspheres with λ/15-sized hole are plotted in Figure 3g, where the green solid lines, the green dashed lines, and the gray solid lines represent the contour lines with a value of 0.5*I_max_*, 0.8*I_max_*, and 0.9*I_max_*, respectively. A comparison of the data in Table 1 and Table 2 shows that, with a hole of λ/15 diameter, the maximum field intensity near the shadow surface of the particle is increased by nearly two times, with a significant decrease in the field localization volume, compared to the unstructured particle.

### 3.2. Microspheres with a Blind Hole

**Figure 4:**
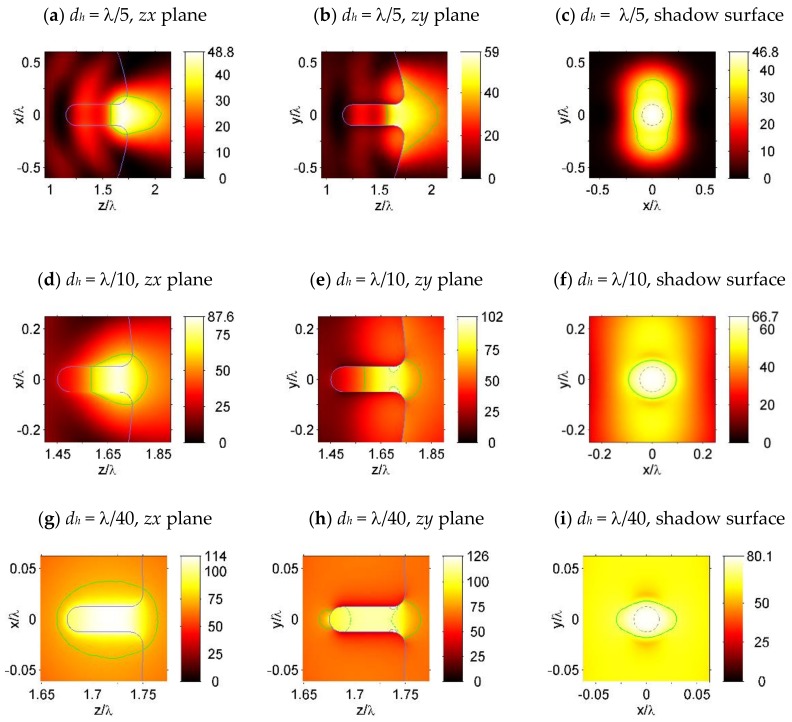
Light intensity (|E|^2^) of the simulated dielectric microspheres with a blind nanohole of the diameter (**a**–**c**) *d_h_* = λ/10, (**d**–**f**) *d_h_* = λ/10, and (**g**–**i**) *d_h_* = λ/40. The sphere diameter and refractive index are set as *D_s_* = 3.5λ and *n* = 1.5, with a hole depth of 3*d_h_*. Subfigures (**a**,**d**,**g**) are plotted in the zx-plane, which is perpendicular to the polarization plane. Subfigures (**b**,**e**,**h**) are plotted in the zy-plane, which is the polarization plane. To indicate the “hot spots” in subfigures (**e**,**h**,**i**), contour lines at the value of 0.5*I_max_*, 0.85*I_max_*, and 0.9*I_max_* are plotted by the green solid lines, the green dashed lines, and the gray solid lines, respectively. Subfigures (**c**,**f**,**i**) show the light intensity along a plane that is 0.001λ from the shadow surface of the sphere and the circles, which are plotted by gray dashed lines, indicating the projection of the hole interface in the shadow surface.

After this, dielectric microspheres with a blind hole at the shadow surface were also simulated and the results are shown in Figure 4. In the simulation, the hole diameters were set to be *d_h_* = λ/5, λ/10, and λ/40, respectively, with a hole depth of 3*d_h_*. The corner radius at the opening of the hole, as well as the fillet radius at the blind end of the hole, were both set to be *r* = *d_h_*/2. From Figure 4a,b, we can see that for a sphere with a hole size of *d_h_* = λ/5, the focusing capability of the sphere is weakened by the hole, compared to a sphere without a hole in Figure 1e,f. When the hole sizes are decreased below λ/10, the focused light spot was mainly confined to the blind hole, as shown in Figure 4c–h. “Hot spots” can be observed in Figure 4e,h, which were located inside the hole and near the opening of the hole in the polarization plane (z–y plane), as indicated by the contour lines at the value of 0.85*I_max_* and 0.9*I_max_*.

## 4. Discussion

**Figure 5:**
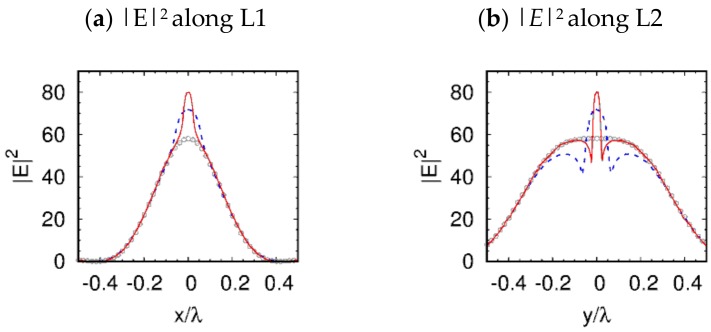
Light intensity along the two imaginary cutting lines L1 and L2, which lie on the shadow surface of the simulated microspheres. L1 is defined as *y* = 0 and *z* = *D_s_*/2 + 0.001λ, which is parallel to the x-axis and perpendicular to the polarization direction. L2 is defined as *x* = 0 and *z* = *D_s_*/2 + 0.001λ, which is parallel to the y-axis, as well as the polarization direction. The gray circles, the blue dashed lines, and the red solid lines indicate the light intensity of the 3.5λ-diameter microsphere without hole, the microsphere with a through hole of λ/15 (see Figure 3e–h), and the microsphere with a blind hole of λ/40 (see Figure 4g–i), respectively.

The effect of 3D-field localization inside and near the open boundary of a hole, which is clearly visible in Figure 3 and Figure 4, is similar to the horizontal slot wave-guide in 2D dimensions [18], as a result of this slot concentrating on the optical energy in the low refractive index region of the wave-guide [19]. It should be noticed that in Reference [18], the refractive index contrast was found to be more than 2. Moreover, in our case, the hole was surrounded by dielectric material with axial symmetry, and the boundaries of such a three-dimensional hole structure were essential for the field localization and enhancement, as is shown in Figure 3 and Figure 4. Furthermore, the high E-field confinement, and the large discontinuity in the air hole, is clearly visible in Figure 5. The E-field confinement in the air hole is dictated by the dielectric refractive index contrast [18,19]. It must be mentioned that, in our case, unlike the slot-wave-guides, the field became localized only near the shadow surface of the particle and not along the entire hole. The observed field enhancement effect was analogous to the lens effect. This allowed us to choose the length of the hole, which was not necessary to be equal to the diameter of the particle, but only a part of it (not the through hole). For example, this could be the length of the field localization region inside the particle, near the shadow surface.

In addition, the proposed nanohole-structured sphere had several unique properties. For example, it could produce a high-field intensity and optical power in relatively low-index materials (air), at levels that cannot be achieved through a conventional PNJ produced by spheres with the same diameter [2,3,4]. This property allowed for highly efficient interactions between materials and the fields, in the hole. Moreover, it could produce a strong field confinement which is localized in a nanometer-sized hole, obtaining a resolution that is comparable to the nanohole size, beyond the diffraction limit. Thus, such nanohole-structured mesoscale particles can be used to significantly enhance the efficiency of the near-field probes, and to increase the sensitivity of the compact optical sensing devices.

## 5. Conclusions

In this work, a nanohole-structured dielectric mesoscale microsphere for a subwavelength-scale light focusing and confinement has been proposed. Numerical simulations showed that light can be focused and confined, considerably, in the nanohole that is located at the shadow surface of the dielectric microparticles, achieving a resolution beyond the solid immersion diffraction limit (λ/2*n*) [20], with a plane wave illumination. The introduction of a nanohole on the shadow surface of a dielectric particle, allows us to “compress” the field localization characteristic of a photonic nanojet, to the size of this nanohole. The proposed nanohole-structured microparticles are important for advancing the particle-lens-based super-resolution technologies, including sub-diffraction imaging, interferometry, surface fabrication, enhanced Raman scattering, optical tweezer, and so on.

## Figures and Tables

**Table 1:** Light focusing properties of the microspheres with a fixed refractive index of *n* = 1.5 and different sphere diameters (*D_s_*).

*D_s_*	*S_x_*	*S_y_*	*S_z_*	*V*	*I_max_*
1.5λ	0.33λ	0.77λ	0.50λ	0.057λ^3^	16.1*I*_0_
2.5λ	0.32λ	0.78λ	0.43λ	0.055λ^3^	35.5*I*_0_
3.5λ	0.35λ	0.74λ	0.49λ	0.051λ^3^	61.1*I*_0_
4.5λ	0.50λ	0.81λ	0.81λ	0.173λ^3^	57.4*I*_0_

**Table 2:** Light focusing properties of the simulated nanohole-structured microspheres with a sphere diameter of *D_s_* = 3.5λ, refractive index of *n* = 1.5, and a through hole of *d_h_* = λ/15 in diameter.

*D_s_*	*S_x_*	*S_y_*	*S_z_*	*V*	*I_max_*
λ/5	0.39λ	0.81λ	0.63λ	0.064λ^3^	48.6*I*_0_
λ/10	0.25λ	0.42λ	0.27λ	0.0033λ^3^	93.5*I*_0_
λ/15	0.20λ	0.14λ	0.25λ	0.0012λ^3^	112*I*_0_
